# Supplementary material for: STAT1 deficiency predisposes to spontaneous otitis media
Source: PLoS One. 2020 Sep 29;15(9):e0239952. doi: 10.1371/journal.pone.0239952 (PMC7523960; doi:10.1371/journal.pone.0239952)
Supplement: Attachment — Submitted filename: Response to Reviewers.docx [file pone.0239952.s001.docx]

**Point-by-point reply**

Answer to the Reviewers

We thank the Reviewers for their valuable comments and constructive suggestions on our manuscript. We address their questions and comments in a point by point reply.

**Reviewer #1**: This is a very nicely presented manuscript with excellent basic science and clinical significance. The discovery that STAT1 deficiency could be a factor in otitis media is important for clinicians seeking to determine factors associated with otitis media in patients.
ABR data are nicely displayed and discussed. Histopathology samples are of high quality and support the conclusions of the authors.

Thank you for your time revising the manuscript and encouraging comments.

Some minor questions should be addressed by the authors:
Why were the ABR thresholds better in homozygous mice at 18 weeks compared to those obtained at 12-13 weeks? Do the authors think that the inflammation had spontaneously resolved, allowing partial recovery of hearing?

We thank the reviewer for this important comment. Yes, we think that inflammation had spontaneously resolved in some cases of the group of 9 and 18 weeks leading to an improvement in histopathology and hearing. The following information has been included in the discussion section and is also part of the answer to Reviewer 2, point 3).

“All STAT1-KO animals of 12-13 weeks presented hearing impairment suggesting a 100% incidence, while STAT1-KO mice of 9 and 18 weeks showed hearing impairment in 3 out of 8 and 7 out 13, respectively, suggesting a moderate incidence ranging from 37.5-54%. Similar high incidence of hearing loss of 90% has been also found in *Enpp1^asj/asj^* mice [3]. We might expect that the incidence of hearing impairment increases with age as previously shown [25]. The ABR thresholds in C3H/HeJ and *Enpp1^asj/asj^* mice also increased with age [3, 9]. However, it was not always the case in STAT1-KO mice. From our histopathology data, the distribution of cases with spontaneous inflammation in STAT1-KO mice between the age groups was variable. Moreover, it seems that spontaneously resolution of inflammation occurred in some STAT1 mice. These findings suggest that at the time point of ABR measurements, the knockout mice of 12-13 weeks were in a stage of chronic inflammation, while the other mice groups were at different stages of inflammatory process or resolved inflammation. Further studies are needed to clarify the role of STAT1 on hearing using the same experimental group of mice over a long period of time. (lines 346-360)

Why was the score pathology lower in homozygous STAT1 deficient mice at 18 weeks compared with 9 and 12 weeks? Do the authors think that the inflammation had spontaneously resolved resulting in improvement in histopathology?

Yes, we think that inflammation had spontaneously resolved in some cases resulting in improvement in histopathology. Following this comment, we have included this information in the discussion section of the revised manuscript.

Discussion section: “Genotype differences had been observed in *Tgif* mutant mice, while homozygous mice demonstrated high incidence of otitis media, heterozygous revealed chronic otitis media with low penetrance [8].In our study, difference in average pathology scores between genotypes was visible in the group of 12-13 weeks that also had shown high incidence of hearing loss. While the average between the genotypes in the other age groups did not reach significance. The pathology scores were scattered and shown more variability then those of the wild type mice. The scattering values observed in knockout mice might reflect different stages and magnitudes of inflammation. Otitis media often resolves spontaneously, but recurrent and otitis with effusion are longer lasting; and thereby can causes more damage in the ear. Barlett *et al*. suggested that *Splunc1^-/-^* mice experienced recurrent episodes of otitis as they observed that the MEC underwent frequent remodeling [6]. The mice groups in this study may indeed contain individuals with spontaneous resolved inflammation that improved histopathology and allowed partial recovering of hearing. While those mice with long period of inflammation might progressively be affected leading to irreversible damage and permanent hearing loss. The STAT1-KO mice seem to be susceptible to otitis media, but we cannot exclude that other factors contribute to inflammation in mice, as the otitis media is a multifactorial disease [26].” (Lines 387-403)

Also, English grammar needs some minor corrections.

Following this suggestion, we have checked and improved the manuscript.

**Reviewer #2**: STAT1 deficiency predispose to spontaneous otitis media.
In this manuscript, Bodmer et al, investigate the consequences of STAT1 deficiency on hearing and inflammation in the inner and middle ear. Using STAT1+/- and STAT1 knockout mice previously characterized, they examined hearing abilities by measuring auditory brainstem response (ABR) at different ages. They also investigate the ear histology, histopathology, as well as structural abnormalities in STAT1 KO mice. They conclude that STAT1 deficiency increases susceptibility to otitis media through spontaneous inflammation.
Overall, the manuscript will benefit from a careful editing of introduction and results sections. Discussion needs to be rewritten to highlight the findings from the current study. Comments are detailed below:

We sincerely thank the Reviewer for its detailed comments and valuable suggestions that help us to improve the manuscript. We have updated the manuscript according to the your and Reviewer 1 suggestions.

Major points:
1)- In line 86 of Material and Methods section, the authors wrote: “ABR thresholds were measured in STAT1 mice aged 3 weeks (WT, n=1; heterozygote, n=7)”. However, in line 160 of the Results section, they mention: “ABR thresholds between WT (n=4 ears) and heterozygotes (n=14 ears) mice were similar.” Can authors clarify the number of WT mice used at 3w?

We apologize for this error. The number of WT mice is 1 and number of ears is 2. We have corrected the number accordingly. (Line 165)

2)- Line 241: “The onset of otitis media seems to start later than 3 weeks as the hearing impairment has not been observed at this young group of mice.” Comparing the ABR measurements in only one WT mouse to 7 heterozygotes is not sufficient to make this conclusion. Can authors provide ABR measurements for more WT mice at 3 weeks?

We thank the reviewer for this comment. In order to clarify our observations, we have now rewritten our sentence as follows:

“We observed hearing impairment in our mice at the age of 9 weeks and not at the age of 3 weeks. Although the ABR thresholds were low and similar in wild type and heterozygous mice at 3 weeks of age; we cannot rule out that homozygous mice might have showed hearing impairments. The timing of the elevation of ABR thresholds seems to differ between knockout mice. Tian et al found that *Enpp1^asj/asj^* mice exhibited normal ABR thresholds at 3 weeks and higher threshold by 6 weeks of age [3]. While C3H/HeJ mice presented normal thresholds at 3 months and started to show elevated thresholds at 5 months [9]. The *Tgif* mutant mice at 2 months of age showed higher average ABR thresholds as compared to wild type. Already at the age of 1 month, the homozygous *Tgif* mice presented reduced Preyer’s reflex [8]. “ (Lines 332-341)

We would provide additional ABR measurements, but unfortunately, due to space limitations in our facility, our research group does not have STAT1 wild type animals at 3 weeks. We are backcrossing the STAT1 from mixed (C57BL/6-129/SvEV) to C57BL/6J background and therefore we do not hold STAT1 mice with mixed background. We have the possibility to get from other research group wild type mice for breeding, however we feel that the overall message of the manuscript will not substantially change with additional data of wild type animals.

3)- Figure 1: At 9 weeks, homozygotes only have a moderate hearing loss and significant hearing impairment is only observed in heterozygotes. Whereas at 12-13 weeks, homozygotes have a significant increase in ABR thresholds. At 18 weeks, there is no significant difference in ABR thresholds between WT, heterozygotes and homozygotes. Can authors discuss this observation and its significance? Do they expect similar ABR thresholds at 18 weeks?

We thank the reviewer for this important comment. We observed that the ABR thresholds were variable in the knockout mice. For better visibility and differentiation of our samples, we added new labels in the Fig 1; those single points showing bilateral and unilateral hearing impairment are now labeled in red and blue, respectively. We include the following paragraph in the discussion section of the revised manuscript to address this point:

“The degrees of hearing impairment in our knockout mice were very variable between the age groups and genotypes. Mice of 12-13 weeks and heterozygous mice of 9 weeks displayed significantly higher hearing thresholds compared to wild type. However, the average of ABR thresholds between the genotypes of other mice groups did not always reach significance. When we further analyze individual mouse in each group, we observed that there was an increased incidence of hearing impairment in knockout mice than in the wild type mice. All STAT1-KO animals of 12-13 weeks presented hearing impairment suggesting a 100% incidence, while STAT1-KO mice of 9 and 18 weeks showed hearing impairment in 3 out of 8 and 7 out 13, respectively, suggesting a moderate incidence ranging from 37.5-54%. Similar high incidence of hearing loss of 90% has been also found in *Enpp1^asj/asj^* mice [3]. We might expect that the incidence of hearing impairment increases with age as previously shown [25]. The ABR thresholds in C3H/HeJ and *Enpp1^asj/asj^* mice also increased with age [3, 9]. However, it was not always the case in STAT1-KO mice. From our histopathology data, the distribution of cases with spontaneous inflammation in STAT1-KO mice between the age groups was variable. Moreover, it seems that spontaneously resolution of inflammation occurred in some STAT1 mice. These findings suggest that at the time point of ABR measurements, the knockout mice of 12-13 weeks were in a stage of chronic inflammation, while the other mice groups were at different stages of inflammatory process or resolved inflammation. Further studies are needed to clarify the role of STAT1 on hearing using the same experimental group of mice over a long period of time. The case of the single wild type mouse with hearing impairment observed in our study might be a case of inborn hearing impairment or acquired later in life.” (lines 342-364)

The following information has been added in the result section:

“In the case of homozygous mice, the average of thresholds was not different between wild type and homozygote, however one mouse (45 dB SPL) out of 4 mice presented higher hearing thresholds than the wild type (Table 1).” (lines 171-174)

“In the group of 18 weeks old mice, the mean value of ABR thresholds was not different between wild type mice and STAT1-KO mice. However, we observed a group of heterozygous and homozygous mice showing high hearing thresholds. Of seven heterozygote mice, three showed bilateral phenotype ranging from profound (> 80 dB SPL) to middle hearing impairment (65 dB SPL) and one unilateral phenotype (50 dB SPL). Of six homozygous mice, one showed bilateral hearing loss (> 80 dB SPL) and two showed unilateral phenotype (65-70 dB SPL). In addition, one out of five WT mice showed bilateral hearing loss (50-60 dB SPL).” (lines 178-185)

In addition, we have included a table with the numbers of mice presenting hearing impairment (affected) and total examined mice in each group.

Table 1 caption: “Number of mice examined. The affected mice included those mice showing unilateral and bilateral hearing impairment.” (196-199)

4)- Can authors comment on the absence of homozygotes in 3 weeks, as well as heterozygotes in 12-13 weeks?

Our first measurements were performed in the group of 12-13 weeks, where we observed hearing impartments in STAT1-KO mice. We decided to evaluate hearing sensitivity in mice at different ages. Due to space limitations in our animal facilities, we had few heterozygotes breeding pairs. We performed ABR measurements in the available litters and afterwards we determined the genotype of each mouse. This may clarify the different numbers of each genotype and missing genotypes. The following sentence has been included in the material and methods section for clarification purposes:

“The genotypes of the mice were determined after ABR measurements, except for the group of 12-13 weeks that was genotyped before ABR measurements.” (lines 87-88)

5)- Line 185: “STAT1-KO mice presented from moderate to very significant degree of inflammation in the middle and inner ear at 9, 12-13 and 18 weeks mice.” In figure 1A, at 9 weeks there is no difference in ABR measurements between homozygotes and WT. Can authors discuss why they observe an inflammation without an impairment in hearing?

We thank to the reviewer for this insightful comment and would like to clarify this information. We did not observe inflammation in STAT1-KO mice without an impairment in hearing. In figure 1A we have shown hearing thresholds in different genotypes, while in figure 5A we have shown the correlation between inflammation and ABR thresholds. We observed a single wild type mouse showing in one ear middle inflammation and normal ABR threshold (Fig 5A). The discussion of this case is giving in point 7). In order to clarify the relationship of hearing impairment and inflammation, we have improved the paragraph in the result section as following:

“All wild type mice presented normal tympanic membrane, normal round window membrane and clear middle ear cavity (MEC) with thin epithelium, except from one mouse of 18 weeks old with normal hearing. This mouse presented in one ear a middle inflammation with light middle and inner ear effusion as well as tissue proliferation. STAT1-KO mice of 9 and 13 weeks with unilateral or bilateral hearing impairment presented from moderate to high levels of inflammation in the affected ear. While those mice with normal hearing did not show signs of inflammation. STAT1-KO mice at 18 weeks presented different situations. All heterozygous mice with high ABR thresholds presented sign of inflammation, except one mouse that presented bilateral hearing loss with one ear affected and the other ear with almost no inflammation. Two homozygous mice with unilateral hearing impairment did not present signs of inflammation in the affected ears. All other STAT1-KO mice with high ABR thresholds presented sings of inflammation, while those mice with normal ABR threshold showed no inflammation signs. Examples of middle ear inflammation in STAT1 mice of all groups are shown in Fig 2.” (Lines 209-223)

6)- Can the authors mark the area with differences between KO and WT on Figure 2. Also, it will make it easier for the readers to have the panels labeled with genotypes, age, as well as high or low ABR thresholds.

We thank the reviewer for this observation of additional labeling. Therefore, the panels have been labeled with genotypes, age and their respective ABR thresholds.

7)- Line 210: “The final total pathology scores for all examined groups (9, 12-13 and 18 week old mice) have shown good correlation with ABR thresholds”. Please refrain from using terms such as “good correlation”, and report the correlation coefficient number instead. The correlation and whether it is meaningful can be discussed in the discussion section.

We thank the reviewer for this important recommendation that help us to improve the presentation and discussion of our findings. Following this recommendation, we have refrained the sentence in the result section and add new text in the discussion section as follow:

Result section: “Individual samples with high ABR thresholds are located further to the right and those with high pathology scores are further up on the graph (Fig 5A). The ABR thresholds and the final total pathology scores for all examined groups have shown strong positive linear relationships achieving correlation coefficients of 0.9, 0.94 and 0.74 for 9, 12-13 and 18 weeks old mice, respectively. This indicated that the ABR thresholds increased as did the score pathologies.” (Lines 283-288)

Discussion section: “The strong positive correlation between ABR thresholds and pathology scores suggested that these variables are associated and increased together. Similar correlations between ABR elevation and presence of middle ear inflammation had been reported [3, 4, 8, 9]. The correlation coefficient of the group of 18 weeks was still strong value, but it was weaker than the other groups of 9 and 12-13 weeks. Animals with low levels of ABR thresholds presented mostly low score pathologies as we might expect. While animals with high hearing thresholds presented mostly high score pathology. However, three mice presented either moderate or high ABR thresholds and low score pathology. In the presence of these outliners the strength of the correlation in the group of 18 weeks was weaker in comparison to the two other groups. These three outliners in the region of high ABR thresholds belong to heterozygous and homozygous mice. Although we did not detect signs of inflammation, the resulting hearing impairment may indeed be the results of interaction of multiple factors, such as genetic background and aging processes. It has been reported that the C57BL/6J mice at the age of 3-6 months start to show elevated hearing thresholds at high frequencies [25]. During the aging processes several changes continuously happen affecting the normal functions in our body in much as in our hearing. This could explain the high ABR thresholds observed in those animals without signs of inflammation. We may therefore expect that the observed correlation will get weaker with the age. Another interesting observation was the presence of one wild type mouse with normal hearing and middle inflammation in one ear. The inflammation in this mouse might have been of short duration or spontaneously resolved and its magnitude was not sufficient to destroy the structure of the auditory organ at the time of ABR measurements.” (Lines 365-386)

8)- Line 221: “We didn’t observe any structural changes between the genotypes of STAT, except for the stria vascularis. The analysis of the thickness of the stria vascularis revealed a significant difference between the STAT1-KO and WT mice in the basal turn of the cochlea (Fig 6).” Please report the SV width number and p value in the text as well as in figure 6.

We thank the reviewer for this important observation. We have added the SV width number and p value in the text. The p value was not added to the figure 6, it was however written in the figure legend. The text has been modified as follow:

“We did not observe any structural changes between the genotypes of STAT1, except for stria vascularis thickness. The analysis of the thickness of the stria vascularis revealed a significant difference between the STAT1-KO (22.5±2.1) and WT (20.6±1.4) mice in the basal turn of the cochlea (p<0.05; Fig 6).” (line 308-312)

9)- Discussion needs to be reorganized and focus on what the authors have found, rather than going through what has been previously published. For example, lines 231 to 241, 242 to 247, 257 to 281, and 284 to 289, are summarizing findings from previous studies. This information would be better placed in the introduction section to give readers context as to the rationale of the manuscript. Authors should discuss their own results in the Discussion section and argue the significance of their findings.

We acknowledge that the discussion was incomplete, we have therefore reorganized the discussion section and included the text from the answers to the major points 3), 7), 10), 11), 12), 14) . We also added the respective information in the introduction section.

10)- Results from this manuscript and previous studies suggest that hearing impairment can be detected from 9 weeks onwards. Can authors discuss why the lowest R2 is observed for STAT1 KO mice at 18 weeks, rather than earlier ages (Figure 5A)? Did they expect the lowest correlation at this age?

We thank the reviewer for this recommendation. We believe that this point is connected with the major point 7). Please, refer to the answer above to major point 7).

11)- Line 214: “There were no differences in score pathology between the genotypes except from the group of 12-13 weeks old mice.” The authors need to discuss the significance of this observation in the Discussion section.

We thank the reviewer for this recommendation. We believe that this topic has previously been considered, please refer to answer 7). In order to clarify this point, we have also modified that sentence in the result section of the revised manuscript.

Result section: “There were no differences in pathology scores between the genotypes except the significant difference that has been found in the group of 12-13 weeks old mice. However, high pathology scores of more than 5 have been observed in individual heterozygous and homozygous mice in all other age groups.” (299-303)

12)- The article by Mizoguchi et al, 2014 (PMID: 24343863) needs to be cited and authors need to discuss the results from this article in the context of their findings in the discussion.

We thank the reviewer for the recommended literature. We have included this article in our discussion section of the revised manuscript. We have included following sentences in the discussion section:

“Early study reported that STAT1-KO mice presented normal production of B-lymphocytes and monocytes in fetal liver as well as the production of T-lymphocytes in neonatal thymus [16]. Moreover, Lee *et al*. reported that lymphocytes of STAT1-KO mice showed enhanced survival and proliferation *in-vitro*, while normal lymphoid homeostasis in STAT1-KO mice was observed [27]. In our study, STAT1-KO mice showed variable degree of inflammatory cell infiltration. Our findings may reflect also an enhanced survival of lymphocytes in those middle ears of STAT1-KO mice with high degree of infiltration. This could be the result of missing immune modulatory activities of STAT1. Similar findings of variation of inflammatory cell content in *Enpp1^asj/asj^* mice had been reported [3]. Interestingly, patients with mutations in STAT1 gene are susceptible to infections including ear infections [17, 19, 21]. Toubiana *et al*. reported patients suffering chronic mucocutaneous candidiasis (CMC) with gain of function (GOF) mutations in STAT1, those patients did not display relevant defects in immune parameters except but low memory B cells and low production of IL-17A^+^ T cells after pathogen stimulation [21]. In addition, Sharfe *et al*. have reported that patients with heterozygous mutations presented progressive loss of T-lymphocytes and reduced STAT1 expression [20]. In contrast, Mizoguchi *et al*. investigated a group of CMC patients and found that 50% patients carried STAT1 GOF mutations. Those patients did not present progressive lymphopenia, but a broad infectious phenotype [19]. It seems that the regulation of an optimal STAT1 activity is important and dysregulation of its activity leads to immune system imbalance.” (lines 404-424)

13)- Examining inflammatory cells such as lymphocytes and macrophages in only conducted for STAT1 KO mice (Figure 3). These experiments need to be performed including WT mice side by side for comparison.

We thank the reviewer for this observation. We would like to point out that we have performed the histopathological examination of all genotypes. Following the recommendation, we have modified the figure 3 and included the pictures from wild type mice to show side by side comparison.

14)- Both gain and loss of function of STAT1 are linked to otitis media (Toubiana et al, 2016 and Sharfe et al 2014). Patients with heterozygous variants in STAT1 present immunodeficiency with progressive loss of lymphocytes, in contrast to what authors have described here. Also, it is reported that siRNA mediated knockdown of STAT1 reduces inflammatory mediators and can be used to treat ototoxicity (Kaur et al, 2011). Can the authors comment on whether this could be a dosage dependent effect? And how their findings relate to previous reports?

We thank the reviewer for pointing this out. The situation of the patients with immunodeficiency is widely discussed in point 12). Referring to the article of Kaur et al, the authors reported that the decrease of STAT1 by means of siRNA attenuated cisplatin-induced inflammation. The authors reported that cisplatin increased STAT1 activity; and after reducing STAT1 by siRNA the increase of inflammatory markers was inhibited in cisplatin treated cells and rat cochlea. Our work is focused instead on spontaneous otitis media, we did not perform any experiments on ototoxicity. While inflammation induced by cisplatin mainly focused on investigating the cell death mechanisms, we aimed to investigate the hearing function in mice lacking STAT1 gene. We believe that our present results are not direct related.

15)- Line 281: “We did not find any tumor in the middle ear of our knockout mice, however STAT1-KO mice presented signs of inflammation.” Where are the experiments supporting their argument on tumors? Authors need to provide these data. These statements need to be included in Results section, and not to be introduced for the first time in Discussion.

We thank the reviewer for pointing this out. Following this and the following recommendations, we have deleted the sentence and edited the paragraph of the discussion section as we did not perform any experiments linked to tumorigenesis.

16)- Line 291: “We could suggest that the roles of STAT1 as tumor suppressor and inflammation regulator were evident in these mice.” Authors have not performed any experiments specifically examining the role of STAT1 in tumorigenesis, and not detecting tumors is not sufficient to make this argument. Authors need to be careful not to over interpret their results.

We agree that this sentence is speculative at this time and we have deleted this sentence and edited our paragraph. Please see 15).

Minor points:
- Line 294 should read “STAT3/STAT5 in absence of STAT1 as observed in cancer cells.”

We apologize for this error. We have modified it accordingly.

- Authors need to maintain a formal tone throughout the manuscript, e.g., line 221, “didn’t” should be replaced by did not.

We thank the reviewer for this remark. We have corrected the text as suggested.

- Figure 4 caption: Rather than repeating the same sentence as included in Results: “While few goblet cells were detected in WT mice, cluster of goblet cells were observed in STAT1-KO mice.”, authors should more specifically describe what the figure is showing and what arrows are indicating.

We thank the reviewer for this recommendation; therefore, we have elaborated this sentence as follow:

“Middle ear sections from wild type and STAT1-KO mice were stained for goblet cells by mucin staining using AB-PAS method. More AB-PAS-positive cells were found in the mucosal layer of the MEC of STAT1-KO mice than in wild type, as indicated by arrows.” (lines 270-273)
